# Overexpression of Ribosomal RNA in the Development of Human Cervical Cancer Is Associated with rDNA Promoter Hypomethylation

**DOI:** 10.1371/journal.pone.0163340

**Published:** 2016-10-03

**Authors:** Hong Zhou, Yapei Wang, Qiongying Lv, Juan Zhang, Qing Wang, Fei Gao, Haoli Hou, Hao Zhang, Wei Zhang, Lijia Li

**Affiliations:** 1 State Key Laboratory of Hybrid Rice, College of Life Sciences, Wuhan University, Wuhan, China; 2 Department of Gynecology and Obstetrics, Zhongnan Hospital, Wuhan University, Wuhan, China; Georgetown University, UNITED STATES

## Abstract

The ribosomal RNA (rRNA) gene encodes rRNA for protein synthesis. Aberrant expression of the rRNA gene has been generally observed in tumor cells and levels of its promoter methylation as an epigenetic regulator affect rRNA gene transcription. The possible relationship between expression and promoter methylation of rDNA has not been examined in human clinical cervical cancer. Here we investigate rRNA gene expression by quantitative real time PCR, and promoter methylation levels by HpaII/MspI digestion and sodium bisulfite sequencing in the development of human cervical cancer. We find that indeed rRNA levels are elevated in most of cervical intraepithelial neoplasia (CIN) specimens as compared with non-cancer tissues. The rDNA promoter region in cervical intraepithelial neoplasia (CIN) tissues reveals significant hypomethylation at cytosines in the context of CpG dinucleotides, accompanied with rDNA chromatin decondensation. Furthermore treatment of HeLa cells with the methylation inhibitor drug 5-aza-2’-deoxycytidine (DAC) demonstrates the negative correlation between the expression of 45S rDNA and the methylation level in the rDNA promoter region. These data suggest that a decrease in rDNA promoter methylation levels can result in an increase of rRNA synthesis in the development of human cervical cancer.

## Introduction

In eukaryotes, 45S ribosomal RNA (rRNA) gene (rDNA) is arranged in arrays of head-to-tail tandem repeats known as nucleolar organizer regions (NORs) [1–4]. The number of rRNA gene copies varies greatly among organisms from fewer than 100 to more than 10 000. The rDNA clusters in mammals include the intergenic spacer and a pre-rRNA coding region. In human, approximately 400 copies of rRNA genes are distributed along five pairs of acrocentric chromosomes 13, 14, 15, 21 and 22, but only parts of genes are active [5]. The rRNA genes are transcribed by RNA polymerase I (Pol I) to produce a long rRNA precursor that is then processed into the 18S, 5.8S and 28S rRNA molecules [6]. These rRNA molecules finally form the ribosome together with 5S rRNA and the ribosomal proteins [6].

The ribosome serves as the center of biological protein synthesis, and thus the production rate of the rRNA is tightly related to cellular growth and proliferation. The balance between the cell growth and ribosome production is maintained in normal cells by regulation of transcription of rRNA genes at an appropriate level whereas this balance in cancer cells is upset and rRNA synthesis is deregulated [7]. The promoter of rDNA includes a core promoter element and an upstream control element (UCE), which plays an important role in rDNA expression. The rDNAs are efficiently transcribed by RNA polymerase I (Pol I), and a number of factors including UBF, NORC, and SL1/ TIF-1B participate in this process [8–10]. Recent studies have shown that epigenetic mechanisms are involved in modulation of Pol I-directed rDNA transcription [11,12]. DNA methylation at cytosines and histone acetylation are the well-characterized epigenetic markers that regulate gene transcription [13]. The active rDNA is rich in acetylated histones and short of CpG methylation at cytosines in its promoter region whereas the silenced rDNA is often correlated with dense CpG methylation and deacetylated histones [14–16].

Abnormal DNA methylation at the promoter CpG islands is steadily gaining credibility as a common event in human cancers and has been connected with overexpression of rRNA genes [17]. A recent study in a MDS (myelodysplastic syndrome) case suggested that increases in DNA methylation in the rDNA promoter could cause decreased rRNA synthesis that may contribute to defective hematopoiesis and bone marrow failure in some individuals with MDS [18]. A significant hypomethylation of CpGs in the rDNA promoter was observed in human hepatocellular carcinomas, in association with the high level of rRNAs [19]. However, a recent report indicated that rRNA levels were increased in human prostate cancer but not linked to rDNA promoter hypomethylation [20]. Therefore it is very sophisticated for rDNA regulation in different human cancers and exploring of rDNA expression mechanism in more types of cancer is required. The relationship between 45S rRNA gene expression and methylation alteration in the rDNA promoter in clinical cervical cancer specimens has not been investigated yet. In this study, our data showed that the 45S rDNA transcription was increased in the majority of clinical cervical intraepithelial neoplasia (CIN) specimens and HeLa cell lines treated with the methylation inhibitor 5-aza-2’-deoxycytidine (DAC) whereas the rDNA promoter region revealed significant hypomethylation, accompanied with rDNA chromatin decondensation. These results suggested that rRNA synthesis and rDNA promoter methylation at CpG islands were inversely correlated in the development of human cervical cancer.

## Materials and Methods

### Ethics approval and consent

The study has been approved by the ethics committee of Wuhan University. The study has been approved by the People's Hospital of Wuhan University too. Ethics approval, permissions and informed consent were obtained from all subjects. We have obtained consent to publish from the participant to report individual patient data. The cervical cancer samples were obtained from patients after informed and the methods were carried out in accordance with the approved guidelines.

### Consent for publication

Written informed consent for publication of their clinical details and/or clinical images was obtained from the patients. A copy of the consent form is available for review by the Editor of this journal.

### Patient samples

Ten patients with cervical intraepithelial neoplasia participated in this study. The ages of patients are from 42 to 72. The ten cervical cancer patients had received operation during 2013–2014 at Gynecological Department in the People's Hospital of Wuhan University, including two CINII patients, two CINIII patients and six CIS patients. All diagnoses were confirmed by pathologic examination and none of them had received radiotherapy or chemotherapy before operation. The primary tumors were clinically classified as International Federation of Gynecologists and Obstetricians stage CIN. Each sample was divided into two portions: one portion was immediately frozen and stored at -80°C; the other was fixed in buffered formalin embodied in paraffin for routine pathologic examination.

### Cell culture

The human cervical cancer HeLa cells were incubated (37°C and 5% CO_2_) in DMEM (Hyclone, Thermo Scientific, USA) supplemented with 10% FBS (Hyclone, Thermo Scientific, USA). After six hours of treatment with 50 μM DAC (A119533, Aladdin, USA), cells were harvested and DNA and RNA were extracted according to manufacturer’s instructions (Invitrogen, Carlsbad, CA, USA).

### Quantitative real-time PCR assay

Total RNAs were isolated using TRIzol reagent (Invitrogen, Carlsbad, CA, USA) from frozen tissues in -80°C. The purified RNA was reverse-transcribed to cDNA by using Revert Aid First Strand cDNA Synthesis Kit (Fermentas, Burlington, ON, Canada). Quantitative real-time PCR was performed using a StepOne Plus real-time PCR system (Applied Biosystems, Carlsbad, USA) in the presence of SYBR Green Real-time PCR Master Mix (TOYOBO, Tokyo, Japan). The amplification conditions were as follow: 94°C for 2 min, followed by 40 amplification cycles at 94°C for 5 s, 56°C for 15 s and 72°C for 20 s. Fluorescence data were acquired at the 72°C step and during the melting-curve program. Quantitative real-time PCR were repeated three times for each sample from three independent experiments. The human *GAPDH* gene was used as a quantitative control for the amplified product. The RT-PCR primers used were: pre-rRNA: 5'- GAA CGG TGG TGT GTC GTT-3' (sense), 5'- GCG TCT CGT CTC GTC TCA CT-3' (antisense) [20]; *GAPDH*: 5'-CCC CTT CAT TGA CCT CAA CTA CAT-3 (sense), 5'-CGC TCC TGG AAG ATG GTG A 3’ (antisense) [18].

### Fluorescence in situ hybridization (FISH)

We obtained an amount of cervical epidermal cells by smears, and then preparation of interphase spread was performed according to standard procedures as below. After centrifugation, cells were washed with PBS and treated with hypotonic solution (0.075M KCl) at 37°C for 30 min. Cells were immediately fixed by Carnoy’s fixative (methanol/acetic acid 3:1). Fixed cell suspension was dropped onto precleaned cold, wet microscope slides. The slides were air-dried overnight and stored in -20°C until analysis. The slides were denatured in a solution (70% formamide in 2×SSC) at 81°C for 3 min. After that, the slides were dehydrated in the graded ethanol series of 75%, 95% and 100% for five minutes each and air-dried. Simultaneously, 1–2 mg/ml probes and 1 mg/ml sheared salmon sperm DNA were pre-mixed in 2×SSC with 10% dextran sulphate and 50% deionized formamide, and denatured at 75°C for 5 min before cooled with ice water immediately. After that, the slides were incubated in denatured hybridization solution for overnight at 37°C. Digoxigenin-labeled probes were detected and amplified with sheep-anti-digoxin-FITC (Roche, Lewes, UK) and rabbit-anti-sheep-FITC (Roche, Lewes, UK) respectively. Each immune reaction was performed for 1 h at 37°C before washing with 1×PBS of 5 min each for three times at room temperature in the dark. Finally, Nuclei was counterstained with premixed DAPI at 1 μg/ml (Sigma, St. Louis, MO, USA), and then observed under an Olympus BX51 fluorescence microscope with appropriate filters for DAPI, Cy3 and FITC respectively. Images obtained using a CCD monochrome camera Sensys 1401E were pseudo-colored and merged using the software Metamorph imaging system (Universal Imaging Corp., PA, USA. version 4.6.3), and final images were edited using Adobe Photoshop 9.0 software.

### DNA isolation and site-specific methylation by HpaI/MapII

Genomic DNA was extracted from frozen CIN and noncancerous tissues obtained from the patients above, according to the manufacturer’s protocol (Beyotime, D0061, Shanghai, China). Then DNA samples were separately digested with HpaII and MspI (Thermo, Dalian, China) according to the instruction of manufacturer, and then detected by RT-PCR. The primers were 5'-TCC GTG TGT GGC TGC GAT-3' (sense) and 5'-CAG GAC AGC GTG TCA GCA AT-3' (antisense).

### Sodium bisulfite modification

A total of 1–2 μg genomic DNA was diluted to 50 μL with distilled H_2_O, denatured in 5.5 μL 3 mol/L NaOH at 55°C for 30 min, then added with 30 μL 10 mmol/L hydroquinone and 520 μL 3.6 mol/L sodium bisulfite, and incubated at 55°C for 12 h with mineral oil to prevent evaporation. Samples were dissolved in 20 μL water and stored at -20°C after purified the modified DNA using Wizard DNA Purification System. The primers for the bisulfite modified promoter of 45S rDNA were 5'-GTT TTT GGG TTG ATT AGA-3' (sense) and 5'-AAA ACC CAA CCT CTC C-3' (antisense). PCR reactions were performed in a 25 μL reaction system containing 2 μL bisulfite-modified DNA, 2 μL 10×PCR buffer, 2 μL 2.5 mmol/L dNTP Mixture, 0.5 μL HiFi Taq-polymerase, 1 μL of each primer and 12.5 μL H_2_O. PCR conditions were 95°C for 4 min, 35 cycles of 94°C for 30 s, annealing at 50°C for 45 s and 72°C for 45 s, followed by a final extension step at 72°C for 10 min, and then reaction was performed on the LifePro Thermal Cycler (Bioer Biotechnology, Hangzhou, China). Unmethylated cytosines were bisulfite converted to uracil that was then transformed to thymidine after PCR while 5-methylcytosine was not converted by bisulfite and remained as a cytosine after PCR. Because the primers did not contain CpG dinucleotides, methylated and unmethylated sequences were amplified with equal efficiency. The obtained product was recovered, purified and cloned. Plasmid DNA from at least fifteen positive clones was sequenced. To guarantee the complete conversion of bisulfite, only the clone where all cytosine residues in nonCpG dinucleotides had been converted to thymine was used for the analysis.

### Statistical analysis

Each data and error bars were calculated from three independent experiments. Data in this manuscript were analysed for significant differences between the experimental groups and control groups by means of t-test. It was considered statistically significant when *P* < 0.05.

## Results

### The pre-rRNA level was increased in human cervical cancer tissues

**Fig 1:**
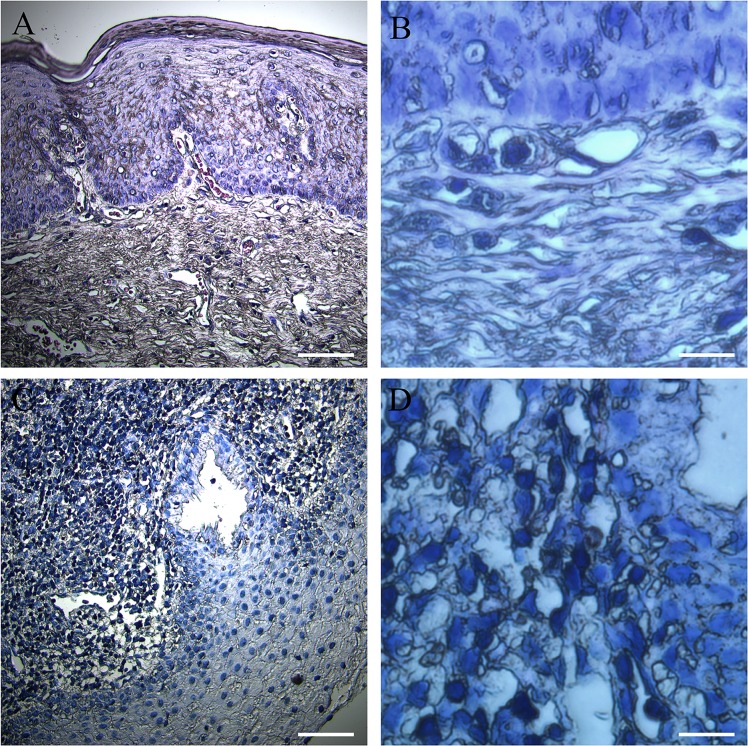
Cervical epithelial cell morphology. (A) A normal cervical epithelial tissue staining pattern; the nucleus of the cervical epithelial cell was stained in blue. Bar = 100 μm. (B) A magnification of a representative non-cancer sample is shown. Bar = 500 μm. (C) A cervical epithelial tissue staining pattern of a cervical epithelial cell abnormality patient; the nucleus which was stained in blue showed obvious abnormalities. Bar = 100 μm. (D) A magnification of a representative CIN sample is shown. Bar = 500 μm.

**Fig 2:**
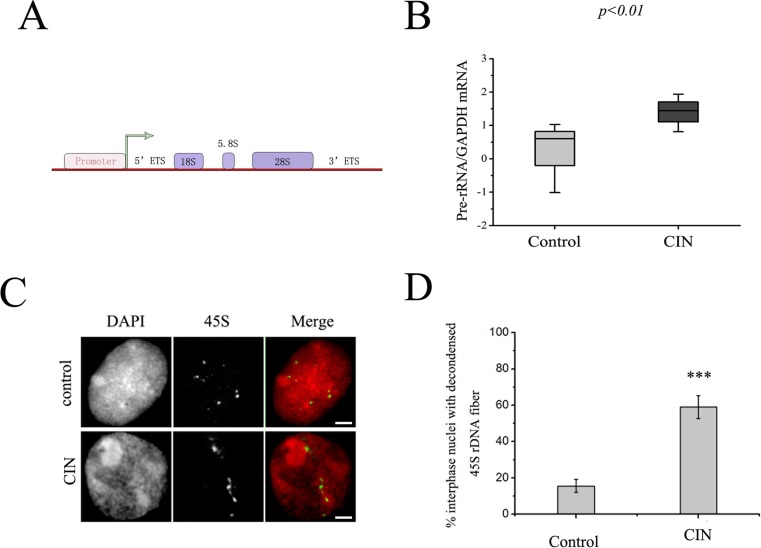
The rRNA level was increased in human cervical cancer samples. (A) Schematic representation of a human 45S rRNA gene. (B) The box plot reveals the relative levels of the rRNA in CIN as compared with the non-tumor tissue. Significance between normal (control) and tumor (CIN) samples was determined using the sign test (the P-value is less than 0.001). (C) Cancer caused aberrant 45S rDNA signal patterns in nuclei. FISH of nuclei with 45S rDNA probes showed spot signals in non-cancer tissues and fiber-like threads unraveled from compacted states in CIN tissues. Bar = 10 μm. (D) Percentages of interphase nuclei with decondensed 45S rDNA fibers in CIN or non-tumor tissues, respectively. Number of evaluated nuclei in each group was 250. Data are expressed as *P<0.05, ***P<0.01, measured by the t-test.

The increased size of nucleoli in cancer cells was observed in almost types of cancer [21]. Indeed, this phenomenon can also be observed in development of human cervical cancer (Fig 1). Along with the development of cancer, the size of the cell was enlarged and atypical nuclear phenomenon occurred, which existed double-core or multi-core. The increased rRNA synthesis occurred very commonly in cancer cells [22]. Therefore, to determine whether the 45S rDNA transcript was elevated in development of human cervical cancer, total RNA was extracted from a series of non-cancerous and cervical precancerous fresh frozen tissue samples which were obtained from ten cervical epithelial cell abnormality patients, and results from real time quantitative reverse transcriptase PCR for the 45S precursor (Fig 2A and 2B) showed that pre-rRNA levels were significantly higher in CIN samples as compared with non-cancerous samples. These data demonstrated that rRNA synthesis was increased in the development of cervical cancer, which is consistent with the knowledge that tumor cells overexpress rRNA species.

### The rDNA showed decondensation in cell nuclei from cervical cancer patients

Next we examined the state of rDNA chromatin in nuclei from cervical cancer patients by fluorescence in situ hybridization (FISH). More than two hundred nuclei were analyzed after a routine nucleus preparation procedure and FISH with 45S rDNA as a probe, and the result indicated that in nuclei there were up to ten signal sites of 45S rDNA but were usually less than ten signal sites, probably due to the three-dimensional structure of the nucleus to hinder to count signals. That is in agreement with the result reported before in human [23,24]. In normal interphase nuclei, a majority of 45S rRNA genes appeared to be compacted into heterochromatic chromocenters and FISH signals occurred as compacted spots. In the presence of CIN, this well-organized structure was disturbed and 45S rDNA formed a diffuse point or a tailing signal look like a beaded fiber unraveled from its normal compact state and spread throughout the nuclei (Fig 2C). The frequency of the nuclei with dispersion was greater than 50% in CIN patients (Fig 2D), which was much higher than in non-cancerous samples.

### The rDNA promoter is hypomethylated in CIN patients

**Fig 3:**
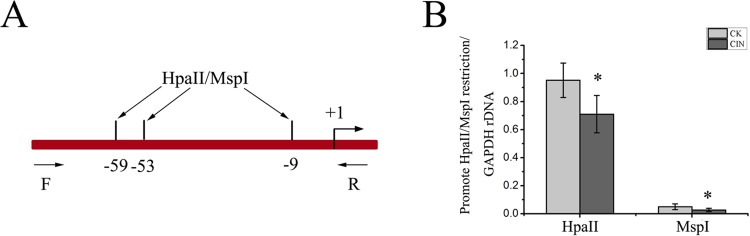
The methylation status of the rRNA gene promoter showed by HpaII/MspI analysis. (A) Schematic of the rRNA gene promoter region reveals the position of quantitative PCR analyzed amplicons and the location of HpaII/MspI restriction enzyme sites. (B) Quantitative PCR analysis of the rDNA promoter using methylation-sensitive HpaII/MspI restriction enzymes. Genomic DNA from CIN and noncancerous tissues was digested with HpaII or MspI and analyzed by PCR. Data are expressed as *P<0.05, measured by the t-test.

**Fig 4:**
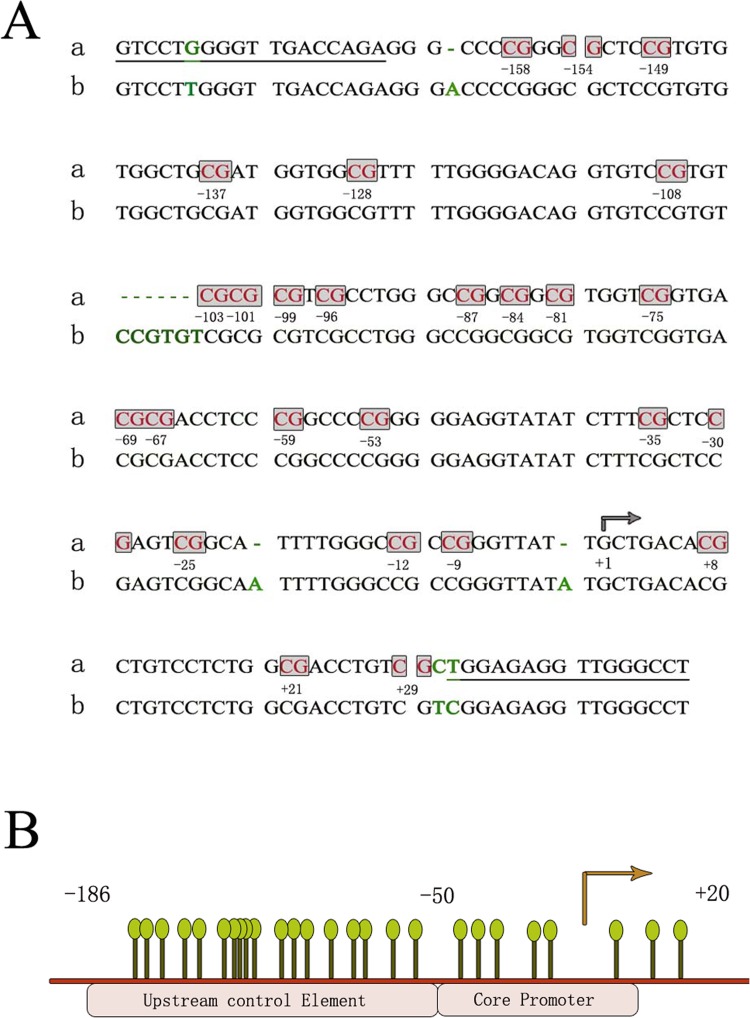
The sequence analysis of the human rRNA gene promoter region. (A) The sequence of the rRNA gene promoter. The rRNA gene promoter sequence (a) was identical for all analyzed CIN and non-tumor tissues and was shown above the published rRNA gene sequence (b). The primer sequences used for sodium bisulfite sequencing are indicated by the underlined regions. CpG dinucleotides are boxed with the position marked relative to the transcription start site (arrowed). Differences with the published rRNA gene sequence, U13369, are highlighted in green. (B) Match rod lines showed the position of CpG dinucleotides on the rRNA gene promoter relative to the transcription start site (arrowed).

**Fig 5:**
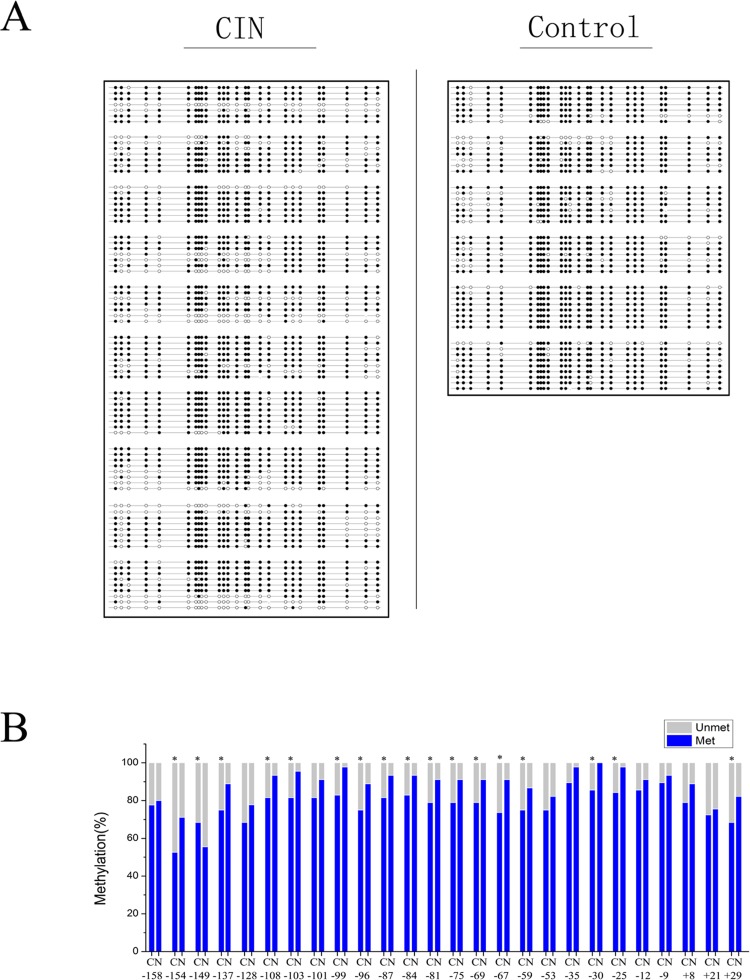
Sodium bisulfite sequencing showed the methylation status of the rRNA gene promoter in clinical CIN tissues as compared with non-cancerous tissues. (A) Sodium bisulfite mapping of the rRNA gene promoter in region of -158 to +29 bp in 10 human CIN tissues and 6 non-cancerous tissues. Each row represents one clone. The filled circle represents methylated CpG dinucleotides and the open circle represents unmethylated CpG dinucleotides. (B) Hypomethylation of rDNA promoter in CIN tissues as compared with normal tissues showed by quantitative analysis of methylation density at each CpG. Theproportion of methylation (blue bar) and unmethylation (grey bar) for each CpG site for CIN tissues (*N* = 10) and non-cancerous tissues (*N* = 6) is showed. C represents the CIN tissue and N represents the non-cancerous tissue. Data are expressed as *P<0.05, measured by the t-test.

To investigate whether the elevated rRNA level in cervical intraepithelial neoplasia was affected by its promoter hypomethylation, we first utilized the isoschizomers HpaII and MspI to determine the methylation level roughly, which recognize the same restriction site (CCGG) but have different sensitivities to certain methylation states of cytosines. DNAs were obtained from cervical intraepithelial neoplasia patients and then digested by HpaII or MspI. The results from the experiments of qPCR using the digested DNA as a template illustrated that theamount of amplified fragments in the rDNA promoter which contains three CCGG restriction sites, was reduced in CIN samples compared with control samples (Fig 3). These indicated that the methylation level was decreased during the development of cervical cancer. Then we applied the bisulfite genomic sequencing method to further determine the methylation level of CpG islands in the rDNA promoter. First, we determined the genotype of the rDNA promoter. The alterations in the sequence of the human rDNA promoter region were appraised by comparsion with the published reference sequence of the human rDNA promoter region (Genebank accession number U13369) (Fig 4A). It was found that two CpG dinucleotides were not observed in the sequenced DNA and three adenines were absent. These discrepancies reflect population-specific variants [25]. The position of 26 CpG dinucleotides on a 229 bp rRNA gene promoter region including the upstream control element and the core promoter sequence relative to the transcription start site was identified (Fig 4B). The rDNA promoter-specific primers were selected to amplify this 229 bp fragment in the promoter region using the sodium bisulfite-treated DNA as a template and then the PCR products were sequenced (Fig 4A). Tissue samples from ten cervical intraepithelial neoplasia patients were used to isolate genomic DNA and at least seven available cloning sequences were obtained from pyrosequencing and the methylation level was lower in CIN samples (Fig 5A). The results showed that most CpG sites were significantly demethylated in CIN subjects as compared with the normal, whereas only one CpG was more methylated in CIN subjects relative to controls (Fig 5B).

### Effect of DAC treatment on pre-rRNA expression and promoter methylation

**Fig 6:**
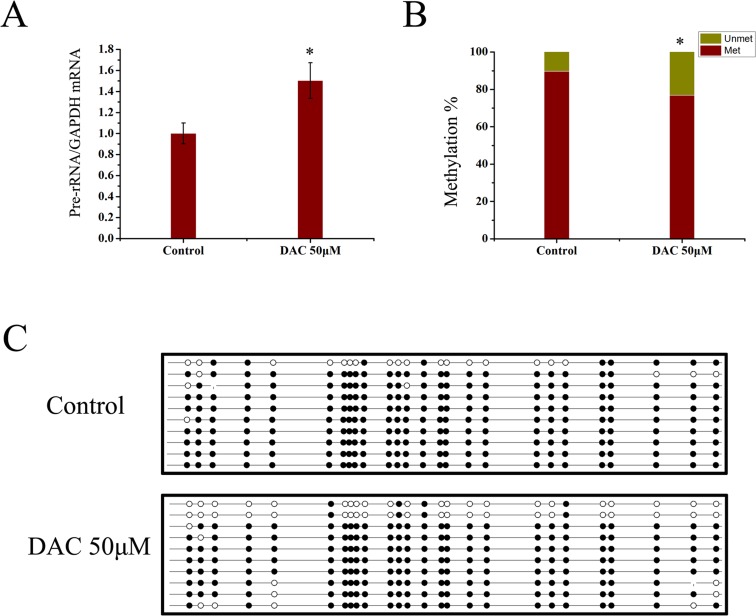
Effect of DAC treatment on pre-rRNA expression and promoter methylation. (A) The level of expression of pre-rRNA in HeLa cells treated with 50 μM DAC for 6 hours is shown relative to that in control cells. *GAPDH* was used as an internal control and the samples were run in triplicates. (B) Theproportion of methylation (red bar) and unmethylation (yellow bar) for 26 CpG sites in HeLa cells which were treated or not treated with 50 μM DAC for six hours is showed. (C) The cells were treated or not treated with 50 μM DAC for six hours and the extent of CpG methylation was determined by pyrosequencing at 26 CpG islands spanning the rDNA upstream core element and the core promoter. Data are expressed as *P<0.05, measured by the t-test.

To further confirm whether decreased rDNA promoter methylation is associated with the increase in rRNA synthesis, we treated the cervical cancer HeLa cell line with the drug DAC. Cells were treated with DAC in four different concentrations for six hours, which are 0.05 μM, 0.5 μM, 5 μM, 50 μM and 100 μM. The results showed that treatment with 50 μM DAC led to a significant increase in the expression of pre-rRNA as compared with cells cultured under normal conditions over the same interval (Fig 6A). A decrease in the methylation level in the promoter region was also observed at 26 CpGs in DAC-treated HeLa cells as measured by pyrosequencing (Fig 6B and 6C). This result further demonstrated the negative correlation between the expression of rDNA and the methylation level in rDNA promoter region.

## Discussion

Fast growing and proliferating cells obviously need an increase in protein production and thus it is not surprising that the rRNA level is elevated in most human cancer cells to meet the demand for increased production of ribosomes and for protein synthesis [26]. So far only few clinical cancer samples have been examined for expression and regulation of rRNA genes. DNA methylation in cancer has been received widespread attention. DNA methylation patterns of the genome usually show disruptions in the cancer cells, and DNA methylation is even altered in the promoter for individual gene in different cancers [27–30]. There is no report on the methylation status of 45S rDNA in relation to its expression changes during the multistage pathogenesis of cervical cancer. This study demonstrated that rRNA levels were increased during the development of cervical cancers, accompanied with epigenetic change, which is involved in hypomethylation of the rDNA promoter and DNA decondensation.

A proposal to elucidate the interaction between rRNA gene transcription and DNA methylation is that the rRNA gene transcription level is modulated by changing the number of the active gene which is directly related to DNA methylation. Research demonstrated that the fraction of methylated sequences corresponds to silent rDNA repeats [31]. During embryonic development of *X*. *Laevis*, hypomethylation of the enhancer region of rDNA is associated with the initiation of rDNA transcription [15,32,33]. The rDNA promoter showed decreased methylation levels in live cancer where rRNA transcription was increased [19]. In lung cancer, it was shown that an increase in rDNA expression was depended on demethylation of H3K9me3 [34]. DNA demethylation in human and mouse cells could antagonize transcriptional repression of silent rRNA genes, resulting in increased pre-rRNA levels [15]. Similarly, the rDNA CpG methylation level was higher in ovarian cancer patients with long progression survival than that in patients with short survival, which was explained that rDNA silence might influence production of ribosomes and protein synthesis required for active tumor proliferation and tumor aggressiveness [35,36]. It is generally thought that DNA methylation is capable of repressing transcription by preventing protein factors from binding to methylated DNA [37]. UBF can bind to a positioned nucleosome on unmethylated DNA, but DNA methylation of the UCE region prevents this interaction [15]. However, by contrast, it has also been reported that DNA methylation level was unchanged in prostate cancer although the expression of 45S rDNA was significantly increased [20]. Similar research in breast cancer showed that the expression of rRNA transcripts might not be based solely on promoter methylation [38]. There data suggested that DNA methylation might not an indispensable means in regulation of rDNA gene transcriptional activity. Actually a number of factors were involved in regulation of the expression of rDNA genes such as transcriptional factors, the MYC protein and Polymerase I. rDNA transcription could be increased by modification of transcriptional factors affecting the efficiency of transcription initiation [22]. However, our results showed that the level of the 45S rRNA was elevated in clinical specimens and HeLa cells treated with DAC, accompanied by significant hypomethylation at cytosines in the context of CpG dinucleotides at the 45S rDNA promoter region as compared with control samples. These data suggested that there was a direct link between DNA methylation and rDNA gene transcription at least in the development of cervical cancer, which was in agreement with hypothesis that promoter DNA methylation appears to be a powerful epigenetic mechanism for silencing of rDNA transcription [39]. Thus DNA methylation is involved in rRNA gene transcription dependent on different cancer cells.

Up-regulation of rDNA transcription is thought to be associated with genome instability that is a mechanism of rDNA transcriptional regulation [40]. Many genetic studies proposed that the rDNA was a dynamic region in terms of copy number and showed some typical characteristics of genetic instability such as gene conversion, senescence and damage resistance [41]. Decondensation was observed at the proximal ends of the loci on chromosomes 10 and 11 in *N*. *sylvestris* [42,43]. The rDNA rearrangements were found in most of adult solid tumors [44]. Furthermore our previous studies have shown that the plant 45S rDNA repeats are cytogenetically transcription-dependent as well as replication-dependent fragile sites that are associated with epigenetic alterations and DNA damage [45]. Fragile phenotypes of 45S rDNA were also observed in some human cancer cell lines where rDNA transcription was increased (data unpublished). Our result of FISH showed the ratio of 45S rDNA deconsendation in nuclei was significantly increased in CIN samples, which might be related to active rRNA transcription. High transcriptional activity was shown to retard local chromatin condensation, especially for tandemly repeated genes [46]. In fact, some rDNA units are in the open states while the other rDNA chromatin exists in inactive states in mammalian cells, which are distinguished by the presence or absence of methylated CpG dinucleotides in the rDNA promoter [47–49]. But there is no evidence to demonstrate whether the reason for increased rRNA levels is by changing the rate of transcription per active rDNA unit or by adjusting the number of active rDNA repeats [50]. Some studies suggested that epigenetic alterations at 45S rDNAs that may prevent chromatin fibers from folding into higher-order metaphase chromosomes are indicators of the highly decondensed states [31,45]. The methylated rDNA units are always inactive and condensed heterochromatin that makes them difficulty to recombine. For example, meiotic recombination in *Ascobolus* is greatly inhibited by methylation [51,52]. Similarly, some studies suggested that genomic demethylation may promote genomic instability and increase the risk of cancer in some tissues [53,54]. However more evidences are needed to elucidate which of genomic instability, epigenetic and cancer is the causal factor or consequence.

## Abbreviations

DAC: 5-aza-2’-deoxycytidine
CIN: clinical cervical intraepithelial neoplasia
NORs: nucleolar organizer regions
UCE: upstream control element
Pol I: RNA polymerase I
PCR: polymerase chain reaction
